# Supplementary material for: Protease-Independent Production of Poliovirus Virus-like Particles in Pichia pastoris: Implications for Efficient Vaccine Development and Insights into Capsid Assembly
Source: Microbiol Spectr. 2022 Dec 12;11(1):e04300-22. doi: 10.1128/spectrum.04300-22 (PMC9927490; doi:10.1128/spectrum.04300-22)
Supplement: Supplemental file 1 — Supplemental material. Download spectrum.04300-22-s0001.pdf, PDF file, 0.07 MB [file spectrum.04300-22-s0001.pdf]

Supplementary Materials

PV1 SC6b P1 Sequence

ATGGGAGCTCAGGTTTCATCACAGAAAGTGGGCGCACATGAAAACTCAAATGGAGCGTATGGTGGTTC
TACCATTAATTACACCACCATTAATTATTATAGAGATTCAGCTAGTAACGCGGCTTCGAAACAGGACT
TCTCTCAAGACCCTTCCAAGTTCACCGAGCCCATCAAGGATGTCCTGATAAAAACAGCCCCAATGCTA
AACTCGCCAAACATAGAGGCTTGCGGGTATAGCGATAGAGTACTGCAATTAACACTGGGAAACTCCAC
TATAACCGCACAGGAGGCGGCTAATTCAGTAGTCGCTTATGGGCGTTGGCCTGAATATCTGAGGGACA
GCGAAGCCAATCCAGTGGACCAGCCGACAGAACCAGAAGTCGCTGCATGCAGGTTTTATACGCTAGAC
ACCGTGTCTTGGACGAAAGAGTCGCGAGGGTGGTGGTGGAAGTTGCCTGATGCACTGAGGGACATGGG
ACTCTTTGGGCAAAATATGTACTACCACTACCTAGGTAGGTCCGGGTACACCGTGCATGTACAGTGTA
ACGCCTCCAAATTCCACCAGGGGGCACTAGGGGTATTCGCCGTACCAGAGATGTGTCTGGCCGGGGAT
AGCAACACCACTACCATGCACACCAGCTATCAAAATGCCAATCCTGGCGAGAAAGGAGGCACTTTCAC
GGGTACGTTCACTCCTGACAACAACCAGACATCACCTGCCCGCAGGTTCTGCCCGGTGGATTACCTCC
TTGGAAATGGCACGTTGTTGGGGAATGCCTTTGTGTTCCCGCACCAGATAATAAACCTACGGACCAAC
AACTGTGCTACACTGGTACTCCCTTACGTGAACTCCCTCTCGATAGATAGTATGGTAAAGCACAATAA
TTGGGGAATTGCAATATTACCATTGGCCCCATTAAATTTTGCTAGTGAGTCCTCCCCAGAGATTCCAA
TCACCTTGACCATAGCCCCTATGTGCTGTGAGTTCAATGGATTAAGAAACATCACCCTGCCACGCTTA
CAGGGCCTGCCGGTCATGAACACCCCTGGTAGCAATCAATATCTTACTGCAGACAACTTCCAGTCACC
GTGTGCGCTGCCTGAATTTGATGTGACCCCACCTATTGACATACCCGGTGAAGTAAAGAACATGATGG
AATTGGCAGAAATCGACACCATGATTCCCTTTGACTTAAGTGCCACAAAAAAGAACACCATGGAAATG
TATAGGGTTCGGTTAAGTGACAAACCACATACAGACGATCCCATACTCTGCCTGTCACTCTCTCCAGC
TTCAGATCCTAGGTTGTCACATACTATGCTTGGAGAAATCCTAAATTACTACACACACTGGGCAGGAT
CCCTGAAGTTCACGTTTATGTTCTGTGGATCCATGATGGCAACTGGCAAACTGTTGGTGTCATACGCG
CCTCCTGGAGCCGACCCACCAAAGAAGCGTAAGGAGGCGATGTTGGGAACACATGTGATCTGGGACAT
AGGACTGCAGTCCTCATGTACTATGGTAGTGCCATGGATTAGCAACACCACGTATCGGCTAACCATAG
ATGATAGTTTCACCGAAGGCGGATACATCAGCGTCTTCTACCAAACTAGAATAGTCGTCCCTCTTTCG
ACACCCAGAGAGATGGACATCCTTGGTTTTGTGTCAGCGTGTAATGACTTCAGCGTGCGCTTGTTGCG
AGATACCACACATATAGAGCAAAAAGCGCTAGCACAGGGGTTAGGTCAGATGCTTGAAAGCATGATTG
ACAACACAGTCCGTGAAACGGTGGGGGCGGCAACATCTAGAGACGCTCTCCCAAACACTGAAGCCAGT
GGACCAACACACTCCAAGGAAATTCCGGCACTCACCGCAGTGGAAACTGGGGCCACAAATCCACTAGT
CCCTTCTGATACAGTGCAAACCAGACATGTTGTACAACATAGGTCAAGGTCAGAGTCTAGCATAGAGT
CTTTCTTCGCGCGGGGTGCATGCGTGACCATTATGACCGTGGATAACCCAGCTTCCACCACGAATAAG
GATAAGCTATTTGCAGTGTGGAAGATCACTTATAAAGATACTGTCCAGTTACGGAGGAAATTGGAGTT
CTTCACCTATTCTAGATTTGATATGGAACTTACCTTTGTGGTTACTGCAAATTTCACTGAGACTAACA
ATGGGCATGCCTTAAATCAAGTGTACCAAATTATGTACGTACCACCAGGCGCTCCAGTGCCCGAGAAA
TGGGACGACTACACATGGCAAACCTCATCAAATCCATCAATCTTTTACACCTACGGAACAGCTCCAGC
CCGGATCTCGGTACCGTATGTTGGTATTTCGAACGCCTATTCACACTTTTACGACGGTTTTTCCAAAG
TACCACTGAAGGACCAGTCGGCAGCACTAGGTGACTCCCTTTATGGTGCAGCATCTCTAAATGACTTC
GGTATTTTGGCTGTTAGAGTAGTCAATGATCCCAACCCGACCAAGGTCACCTCCAAAATCAGAGTGTA
TCTAAAACCCAAACACATCAGAGTCTGGTGCCCGCGTCCACCGAGGGCAGTGGCGTACTACGGCCCTG
GAGTGGATTACAAGGATGGTACGCTTACACCCCTCTCCACCAAGGATCTGACCACATATTAG

PV1 SC6b 6xHIS P1 Sequence

ATGGGAGCTCAGGTTTCATCACAGAAAGTGGGCGCACATGAAAACTCAAATGGAGCGTATGGTGGTTC
TACCATTAATTACACCACCATTAATTATTATAGAGATTCAGCTAGTAACGCGGCTTCGAAACAGGACT
TCTCTCAAGACCCTTCCAAGTTCACCGAGCCCATCAAGGATGTCCTGATAAAAACAGCCCCAATGCTA
AACTCGCCAAACATAGAGGCTTGCGGGTATAGCGATAGAGTACTGCAATTAACACTGGGAAACTCCAC
TATAACCGCACAGGAGGCGGCTAATTCAGTAGTCGCTTATGGGCGTTGGCCTGAATATCTGAGGGACA
GCGAAGCCAATCCAGTGGACCAGCCGACAGAACCAGAAGTCGCTGCATGCAGGTTTTATACGCTAGAC
ACCGTGTCTTGGACGAAAGAGTCGCGAGGGTGGTGGTGGAAGTTGCCTGATGCACTGAGGGACATGGG
ACTCTTTGGGCAAAATATGTACTACCACTACCTAGGTAGGTCCGGGTACACCGTGCATGTACAGTGTA
ACGCCTCCAAATTCCACCAGGGGGCACTAGGGGTATTCGCCGTACCAGAGATGTGTCTGGCCGGGGAT
AGCAACACCACTACCATGCACACCAGCTATCAAAATGCCAATCCTGGCGAGAAAGGAGGCACTTTCAC
GGGTACGTTCACTCCTGACAACAACCAGACATCACCTGCCCGCAGGTTCTGCCCGGTGGATTACCTCC
TTGGAAATGGCACGTTGTTGGGGAATGCCTTTGTGTTCCCGCACCAGATAATAAACCTACGGACCAAC
AACTGTGCTACACTGGTACTCCCTTACGTGAACTCCCTCTCGATAGATAGTATGGTAAAGCACAATAA
TTGGGGAATTGCAATATTACCATTGGCCCCATTAAATTTTGCTAGTGAGTCCTCCCCAGAGATTCCAA
TCACCTTGACCATAGCCCCTATGTGCTGTGAGTTCAATGGATTAAGAAACATCACCCTGCCACGCTTA
CAGGGCCTGCCGGTCATGAACACCCCTGGTAGCAATCAATATCTTACTGCAGACAACTTCCAGTCACC
GTGTGCGCTGCCTGAATTTGATGTGACCCCACCTATTGACATACCCGGTGAAGTAAAGAACATGATGG
AATTGGCAGAAATCGACACCATGATTCCCTTTGACTTAAGTGCCACAAAAAAGAACACCATGGAAATG
TATAGGGTTCGGTTAAGTGACAAACCACATACAGACGATCCCATACTCTGCCTGTCACTCTCTCCAGC
TTCAGATCCTAGGTTGTCACATACTATGCTTGGAGAAATCCTAAATTACTACACACACTGGGCAGGAT
CCCTGAAGTTCACGTTTATGTTCTGTGGATCCATGATGGCAACTGGCAAACTGTTGGTGTCATACGCG
CCTCCTGGAGCCGACCCACCAAAGAAGCGTAAGGAGGCGATGTTGGGAACACATGTGATCTGGGACAT
AGGACTGCAGTCCTCATGTACTATGGTAGTGCCATGGATTAGCAACACCACGTATCGGCTAACCATAG
ATGATAGTTTCACCGAAGGCGGATACATCAGCGTCTTCTACCAAACTAGAATAGTCGTCCCTCTTTCG
ACACCCAGAGAGATGGACATCCTTGGTTTTGTGTCAGCGTGTAATGACTTCAGCGTGCGCTTGTTGCG
AGATACCACACATATAGAGCAAAAAGCGCTAGCACAGGGGTTAGGTCAGATGCTTGAAAGCATGATTG
ACAACACAGTCCGTGAAACGGTGGGGGCGGCAACATCTAGAGACGCTCTCCCAAACACTGAAGCCAGT
GGACCAACACACTCCAAGGAAATTCCGGCACTCACCGCAGTGGAAACTGGGGCCACAAATCCACTAGT
CCCTTCTGATACAGTGCAAACCAGACATGTTGTACAACATAGGTCAAGGTCAGAGTCTAGCATAGAGT
CTTTCTTCGCGCGGGGTGCATGCGTGACCATTATGACCGTGGATAACCCAGCTTCCACCACGAATAAG
GATAAGCTATTTGCAGTGTGGAAGATCACTTATAAAGATACTGTCCAGTTACGGAGGAAATTGGAGTT
CTTCACCTATTCTAGATTTGATATGGAACTTACCTTTGTGGTTACTGCAAATTTCACTGAGACTAACA
ATGGGCATGCCTTAAATCAAGTGTACCAAATTATGTACGTACCACCAGGCGCTCCAGTGCCCGAGAAA
TGGGACGACTACACATGGCAAACCTCATCAAATCCATCAATCTTTTACACCTACGGAACAGCTCCAGC
CCGGATCTCGGTACCGTATGTTGGTATTTCGAACGCCTATTCACACTTTTACGACGGTTTTTCCAAAG
TACCACTGAAGGACCAGTCGGCAGCACTAGGTGACTCCCTTTATGGTGCAGCATCTCTAAATGACTTC
GGTATTTTGGCTGTTAGAGTAGTCAATGATCCCAACCCGACCAAGGTCACCTCCAAAATCAGAGTGTA
TCTAAAACCCAAACACATCAGAGTCTGGTGCCCGCGTCCACCGAGGGCAGTGGCGTACTACGGCCCTG
GAGTGGATTACAAGGATGGTACGCTTACACCCCTCTCCACCAAGGATCTGACCACATATCATCACCAT
CACCATCACTAG

PV1 3CD *Pichia*-optimised Sequence

ATGGGGCCTGGGTTTGACTATGCAGTAGCCATGGCCAAGAGAAACATTGTTACAGCAACTACATCCAA
AGGTGAGTTCACAATGTTGGGTGTTCATGATAATGTTGCAATTTTACCAACTCACGCTTCACCAGGCG
AATCAATTGTCATTGATGGGAAGGAGGTGGAGATACTAGACGCTAAAGCTTTAGAGGATCAGGCCGGC
ACCAACTTAGAAATCACCATTATCACATTAAAAAGAAACGAAAAATTCCGAGACATCAGACCACACAT
TCCTACTCAGATTACTGAGACAAACGATGGGGTCCTGATTGTCAATACGTCTAAGTATCCCAATATGT
ATGTTCCAGTTGGAGCTGTAACAGAGCAAGGGTACTTAAACTTGGGTGGTAGACAGACTGCCAGAACT
CTTATGTACAACTTTCCTACAAGAGCTGGTCAGTGCGGTGGAGTGATTACCTGCACAGGAAAGGTCAT
TGGTATGCACGTTGGTGGTAATGGTTCCCACGGTTTCGCTGCCGCTTTGAAGCGTTCTTATTTTACTC
AATCCTCCCAAGGAGAGATCCAGTGGATGAGACCCTCTAAAGAGGTTGGCTATCCCATAATTAACGCC
CCTTCTAAGACTAAACTTGAGCCATCTGCCTTTCATTATGTCTTTGAAGGCGTCAAAGAACCCGCAGT
GTTGACTAAGAATGACCCTAGATTGAAAACGGATTTTGAGGAGGCCATATTTTCTAAATATGTCGGTA
ACAAGATTACCGAAGTTGATGAATACATGAAGGAAGCCGTTGACCATTATGCTGGACAGTTGATGTCT
TTGGACATTAACACTGAGCAGATGTGCCTTGAAGATGCTATGTACGGTACTGATGGTCTGGAAGCTTT
AGATCTGAGTACCTCTGCTGGTTATCCATATGTCGCTATGGGTAAGAAGAAAAGAGATATTCTGAATA
AACAAACTAGAGATACTAAGGAAATGCAAAAGCTGTTGGACACATACGGGATCAATCTTCCTTTGGTG
ACCTATGTAAAAGATGAGCTAAGGTCTAAGACAAAGGTTGAACAGGGTAAGTCTCGATTAATTGAAGC
ATCTTCACTTAATGATTCTGTAGCAATGAGGATGGCCTTTGGTAACCTTTATGCTGCCTTCCATAAAA
ACCCAGGCGTGATCACGGGATCTGCTGTCGGTTGCGATCCCGATTTGTTTTGGTCCAAAATCCCTGTG
CTAATGGAGGAGAAGTTATTTGCCTTTGACTACACTGGCTATGACGCATCATTATCTCCAGCATGGTT
CGAAGCCTTGAAAATGGTCCTTGAGAAGATAGGCTTTGGAGATAGAGTAGATTACATTGATTATTTGA
ATCACTCTCATCATTTATATAAGAATAAGACTTACTGTGTTAAGGGTGGAATGCCTTCTGGTTGTTCC
GGGACATCCATTTTCAATAGTATGATCAACAACTTGATCATAAGAACATTGCTGCTTAAAACGTACAA
GGGTATTGATCTAGATCACTTGAAAATGATCGCTTACGGTGACGATGTCATCGCTTCCTATCCTCACG
AAGTCGACGCATCTCTTCTTGCCCAGAGTGGAAAAGATTACGGACTAACCATGACTCCAGCAGACAAG
AGTGCTACTTTTGAAACCGTGACATGGGAAAACGTGACTTTCTTGAAGAGGTTTTTTAGAGCCGACGA
AAAATACCCATTCTTAATACACCCCGTCATGCCTATGAAGGAAATTCACGAGTCTATCCGTTGGACGA
AAGACCCACGTAACACTCAGGATCATGTGAGGTCTCTATGTCTATTAGCTTGGCACAATGGTGAAGAG
GAGTACAATAAATTCCTTGCAAAGATCAGAAGTGTTCCCATAGGACGTGCATTGTTGTTGCCAGAATA
TTCCACCCTGTACCGTAGATGGCTAGACTCTTTTTAG

PV1 SC6b VP0

ATGGGAGCTCAGGTTTCATCACAGAAAGTGGGCGCACATGAAAACTCAAATGGAGCGTATGGTGGTTC
TACCATTAATTACACCACCATTAATTATTATAGAGATTCAGCTAGTAACGCGGCTTCGAAACAGGACT
TCTCTCAAGACCCTTCCAAGTTCACCGAGCCCATCAAGGATGTCCTGATAAAAACAGCCCCAATGCTA
AACTCGCCAAACATAGAGGCTTGCGGGTATAGCGATAGAGTACTGCAATTAACACTGGGAAACTCCAC
TATAACCGCACAGGAGGCGGCTAATTCAGTAGTCGCTTATGGGCGTTGGCCTGAATATCTGAGGGACA
GCGAAGCCAATCCAGTGGACCAGCCGACAGAACCAGAAGTCGCTGCATGCAGGTTTTATACGCTAGAC
ACCGTGTCTTGGACGAAAGAGTCGCGAGGGTGGTGGTGGAAGTTGCCTGATGCACTGAGGGACATGGG
ACTCTTTGGGCAAAATATGTACTACCACTACCTAGGTAGGTCCGGGTACACCGTGCATGTACAGTGTA
ACGCCTCCAAATTCCACCAGGGGGCACTAGGGGTATTCGCCGTACCAGAGATGTGTCTGGCCGGGGAT
AGCAACACCACTACCATGCACACCAGCTATCAAAATGCCAATCCTGGCGAGAAAGGAGGCACTTTCAC
GGGTACGTTCACTCCTGACAACAACCAGACATCACCTGCCCGCAGGTTCTGCCCGGTGGATTACCTCC
TTGGAAATGGCACGTTGTTGGGGAATGCCTTTGTGTTCCCGCACCAGATAATAAACCTACGGACCAAC
AACTGTGCTACACTGGTACTCCCTTACGTGAACTCCCTCTCGATAGATAGTATGGTAAAGCACAATAA
TTGGGGAATTGCAATATTACCATTGGCCCCATTAAATTTTGCTAGTGAGTCCTCCCCAGAGATTCCAA
TCACCTTGACCATAGCCCCTATGTGCTGTGAGTTCAATGGATTAAGAAACATCACCCTGCCACGCTTA
CAG

PV1 SC6b VP3

GGCCTGCCGGTCATGAACACCCCTGGTAGCAATCAATATCTTACTGCAGACAACTTCCAGTCACCGTG
TGCGCTGCCTGAATTTGATGTGACCCCACCTATTGACATACCCGGTGAAGTAAAGAACATGATGGAAT
TGGCAGAAATCGACACCATGATTCCCTTTGACTTAAGTGCCACAAAAAAGAACACCATGGAAATGTAT
AGGGTTCGGTTAAGTGACAAACCACATACAGACGATCCCATACTCTGCCTGTCACTCTCTCCAGCTTC
AGATCCTAGGTTGTCACATACTATGCTTGGAGAAATCCTAAATTACTACACACACTGGGCAGGATCCC
TGAAGTTCACGTTTATGTTCTGTGGATCCATGATGGCAACTGGCAAACTGTTGGTGTCATACGCGCCT
CCTGGAGCCGACCCACCAAAGAAGCGTAAGGAGGCGATGTTGGGAACACATGTGATCTGGGACATAGG
ACTGCAGTCCTCATGTACTATGGTAGTGCCATGGATTAGCAACACCACGTATCGGCTAACCATAGATG
ATAGTTTCACCGAAGGCGGATACATCAGCGTCTTCTACCAAACTAGAATAGTCGTCCCTCTTTCGACA
CCCAGAGAGATGGACATCCTTGGTTTTGTGTCAGCGTGTAATGACTTCAGCGTGCGCTTGTTGCGAGA
TACCACACATATAGAGCAAAAAGCGCTAGCACAG

PV1 SC6b VP1

GGGTTAGGTCAGATGCTTGAAAGCATGATTGACAACACAGTCCGTGAAACGGTGGGGGCGGCAACATC
TAGAGACGCTCTCCCAAACACTGAAGCCAGTGGACCAACACACTCCAAGGAAATTCCGGCACTCACCG
CAGTGGAAACTGGGGCCACAAATCCACTAGTCCCTTCTGATACAGTGCAAACCAGACATGTTGTACAA
CATAGGTCAAGGTCAGAGTCTAGCATAGAGTCTTTCTTCGCGCGGGGTGCATGCGTGACCATTATGAC
CGTGGATAACCCAGCTTCCACCACGAATAAGGATAAGCTATTTGCAGTGTGGAAGATCACTTATAAAG
ATACTGTCCAGTTACGGAGGAAATTGGAGTTCTTCACCTATTCTAGATTTGATATGGAACTTACCTTT
GTGGTTACTGCAAATTTCACTGAGACTAACAATGGGCATGCCTTAAATCAAGTGTACCAAATTATGTA
CGTACCACCAGGCGCTCCAGTGCCCGAGAAATGGGACGACTACACATGGCAAACCTCATCAAATCCAT
CAATCTTTTACACCTACGGAACAGCTCCAGCCCGGATCTCGGTACCGTATGTTGGTATTTCGAACGCC
TATTCACACTTTTACGACGGTTTTTCCAAAGTACCACTGAAGGACCAGTCGGCAGCACTAGGTGACTC
CCTTTATGGTGCAGCATCTCTAAATGACTTCGGTATTTTGGCTGTTAGAGTAGTCAATGATCCCAACC
CGACCAAGGTCACCTCCAAAATCAGAGTGTATCTAAAACCCAAACACATCAGAGTCTGGTGCCCGCGT
CCACCGAGGGCAGTGGCGTACTACGGCCCTGGAGTGGATTACAAGGATGGTACGCTTACACCCCTCTC
CACCAAGGATCTGACCACATAT

GSG-Porcine Teschovirus 2A

GGAAGCGGAGCTACTAACTTCAGCCTGCTGAAGCAGGCTGGAGACGTGGAGGAGAACCCTGGACCT
